# *Punica granatum *L. (Pomegranate) Extract:* In Vivo* Study of Antimicrobial Activity against* Porphyromonas gingivalis *in* Galleria mellonella* Model

**DOI:** 10.1155/2016/8626987

**Published:** 2016-09-07

**Authors:** Livia Aparecida Procópio Gomes, Lívia Mara Alves Figueiredo, Ana Luiza do Rosário Palma, Barbara Maria Corrêa Geraldo, Kelly Cristine Isler Castro, Luciana Ruano de Oliveira Fugisaki, Antônio Olavo Cardoso Jorge, Luciane Dias de Oliveira, Juliana Campos Junqueira

**Affiliations:** Department of Biosciences and Oral Diagnosis, Laboratory of Microbiology and Immunology, Institute of Science and Technology, Universidade Estadual Paulista (UNESP), Av. Engenheiro Francisco José Longo 777, Jardim São Dimas, São José dos Campos, SP, Brazil

## Abstract

Due to the increase of bacterial resistance, medicinal alternatives are being explored.* Punica granatum* L. is an effective herbal extract with broad spectrum of action and bactericidal, antifungal, anthelmintic potential and being able to modulate the immune response. The aim was to evaluate the antimicrobial activity of pomegranate glycolic extract (PGE) against the periodontal pathogen* Porphyromonas gingivalis* by using* Galleria mellonella* as* in vivo* model. Fifteen larvae were used per group. Injection of high concentration (200, 100, and 25 mg/mL) of PGE showed a toxic effect, leading them to death. A suspension of* P. gingivalis* (10^6^ cells/mL) was inoculated in the left last proleg and PGE (12.5, 6.25, 3.1, and 2.5 mg/mL) were injected into the right proleg. The larvae were then kept at 37°C under the dark. Injection of PGE at any dose statistically improved larvae survival rates. The data were analysed (log-rank test, Mantel-Cox, *P* < 0.05) and showed that all concentrations of PGE (12.5, 6.25, 3.1, and 2.5 mg/mL) presented higher larval survival rates, with significant statistical difference in relation to control group (*P. gingivalis*). In conclusion, the PGE had antimicrobial action against* P. gingivalis in vivo* model using* G. mellonella*.

## 1. Introduction

Nowadays, there are an increasing number of researchers seeking alternatives to antibiotics due to the high number of infections whose agents are resistant to these drugs and the emergence of new pathogenic strains. One of the safe and economical alternatives studied is the use of medicinal plants which have shown some antimicrobial activity [1–4].

The use of medicinal plants in the treatment of diseases is increasing worldwide. According to Veiga Jr. et al. [5], the World Health Organization (WHO) defines medicinal plant as any plant possessing substances which can be used for therapeutic purposes or used in the production of semisynthetic drugs.

A widely studied medicinal plant is* Punica granatum *L. (pomegranate) [2] as it has bactericidal, antifungal properties and can modulate the immune response [1, 6–13]. In fact, its effects on diseases affecting the human oral cavity (e.g., periodontitis) are currently being evaluated [14, 15].

According to Schmuch et al. [14], most microorganisms which colonize the gingival sulcus or the gingival margin are compatible with periodontal health. However, only a subset of species can cause or contribute to the progression of periodontitis.* Porphyromonas gingivalis*, an opportunistic Gram-negative anaerobic bacterium, is considered the main etiological agent of chronic and most aggressive forms of periodontal disease [15, 16].

Models of invertebrates have been used in pathogenicity studies of microorganisms in which the host-pathogen interaction is evaluated in an attempt to develop and test new therapies [17, 18]. The use of invertebrate models offers numerous advantages over the conventional animal experimental model, such as low maintenance costs, possibility of large-scale use, and absence of ethical restrictions [18, 19].

*Galleria mellonella* is an invertebrate model presenting many advantages for human infection studies.* G. mellonella* is the moth caterpillar whose natural habitat is the beehive, feeding on honey, pollen, and beeswax. This insect can be created under a temperature of 25°C to 37°C. Its life cycle includes larval and pupal stages before the final transformation into moth [20, 21]. This invertebrate has a cellular and humoral defense system, with production of antimicrobial peptides and hemocytes by its hemolymph system [4, 22–24].

Since* in vivo* studies are crucial for evaluation of the antimicrobial activities of new therapeutic agents and their modulatory effects on immune response, the aim of this study is to evaluate the efficacy of glycolic extract of pomegranate against* P. gingivalis* infection by using a* G. mellonella *experimental model.

## 2. Materials and Methods

### 2.1. Plant Material

The glycolic extract of pomegranate (200 mg/mL) was donated by the MAPRIC Pharmaceuticals and Cosmetics Company (São Paulo, Brazil). The plant's part used to obtain this extract was the fruit, which contained alkaloids (i.e., pelletierine and isopelletierine) and gallic tannins.

### 2.2. Microorganisms and Culture

A type strain of* P. gingivalis* (ATCC 33277) was used. This strain was obtained from the Laboratory of Microbiology and Immunology of the Institute of Science and Technology, UNESP.

*P. gingivalis* was grown on medium containing fastidious anaerobe agar medium (FAA) plus blood agar and supplemented with 0.1% hemin and menadione, followed by incubation in anaerobic jar at 37°C for 5 to 7 days.

Then, a standardized suspension of 10^7^ cells/mL was prepared by spectrophotometry (660 nm). For virulence analysis in* G. mellonella*, this standardized solution was diluted in phosphate buffered saline (PBS) up to a concentration of 10^6^ cells/mL.

### 2.3. *G. mellonella*

This study employed the methodology described by Mylonakis and Aballay [25] and Fuchs et al. [20].* G. mellonella* was also obtained from the Laboratory of Microbiology and Immunology of the Institute of Science and Technology, UNESP, at final larval stage, with a body weight of approximately 250–300 mg being used for each group. All larvae used in the experiment had clear color and were free of spots and/or dark pigments on their cuticle, which could indicate impairment of the animal due to some infectious process and influence the results of the experiment. The experiment was performed in duplicate.

Initially, the toxicity of the glycolic extract of* P. granatum *L. in* G. mellonella* was assessed before a second assessment of its antimicrobial action. In both steps,* G. mellonella *survival test was used.

### 2.4. Toxicity of Glycolic Extract of* P. granatum *L

Different concentrations of glycolic extract of* P. granatum *L. (i.e., 200 mg/mL, 100 mg/mL, 25 mg/mL, 12.5 mg/mL, 6.25 mg/mL, 3.1 mg/mL, and 2.5 mg/mL) diluted in PBS were inoculated in the last proleg of each larva. For analysis of each concentration, 15 larvae were used, totalizing 105 larvae for toxicity evaluation. The larvae were kept in Petri dish at 37°C in the dark and then 10 *μ*L Hamilton syringes (Hamilton Inc., USA) were used for injections. The number of dead* G. mellonella* was daily recorded for 168 hours (i.e., 7 days) for analysis of the survival curve. The larvae were considered dead when they did not show any movement after being touched. The larvae received no nutrition during the experiments in our study.

After obtaining nontoxic concentrations in* G. mellonella*, they were used for assessment of the antimicrobial action of the glycolic extract of* P. granatum *L. against* P. gingivalis.*

### 2.5. Antimicrobial Action of Glycolic Extract of* P. granatum *L

Standardized suspension of* P. gingivalis* (10^6^ cells/larva) was inoculated in the last proleg of each larva. Four concentrations of glycolic extract of* P. granatum* L. (12.5 mg/mL, 6.25 mg/mL, 3.1 mg/mL, and 2.5 mg/mL) not toxic for the larvae were also injected in the other last proleg. For this test, 150 larvae were used.  Table 1 lists the experimental groups tested.

After inoculations for analysis of toxicity and antimicrobial action of the glycolic extract of pomegranate, the larvae were kept in Petri dish at 37°C in the dark. The analysis of the survival curve was performed, as described above.

### 2.6. Statistical Analysis

Survival and death curves of* G. mellonella* were plotted and statistical analysis was performed by using the log-rank test (Mantel-Cox) and the GraphPad Prism statistical software. Significance level was set at *P* < 0.05.

## 3. Results

### 3.1. Toxicity of Glycolic Extract of* P. granatum* L

**Figure 1:**
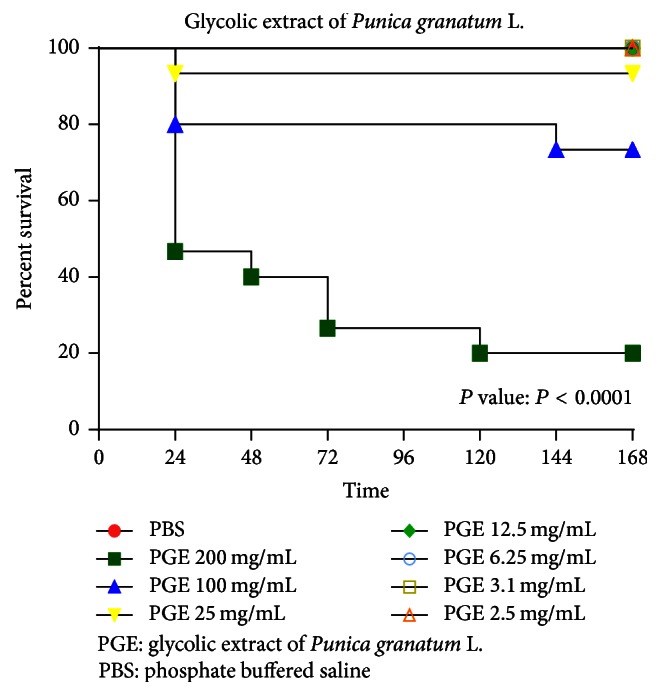
Survival curve of* G. mellonella* with glycolic extract of pomegranate. Different concentrations of PGE (200 mg/mL, 100 mg/mL, 25 mg/mL, 12.5 mg/mL, 6.25 mg/mL, 3.1 mg/mL, and 2.5 mg/mL) diluted in PBS were inoculated in the last proleg of each larva. Fifteen larvae were used per group. The number of dead* G. mellonella* was daily recorded for 168 hours for analysis of the survival curve. Injection of high concentration (200, 100, and 25 mg/mL) of PGE showed a toxic effect, leading* G. mellonella* to death.

The glycolic extract of pomegranate at concentrations of 200 mg/mL, 100 mg/mL, and 25 mg/mL proved to be toxic to the larvae, leading them to death, and they survived at concentrations of 12.5 mg/mL, 6.25 mg/mL, 3.1 mg/mL, and 2.5 mg/mL (Figure 1). The PBS group showed no death after 168 hours in all experiments, thus demonstrating no interference with the obtained results.

### 3.2. Antimicrobial Action of Glycolic Extract of* P. granatum *L

**Figure 2:**
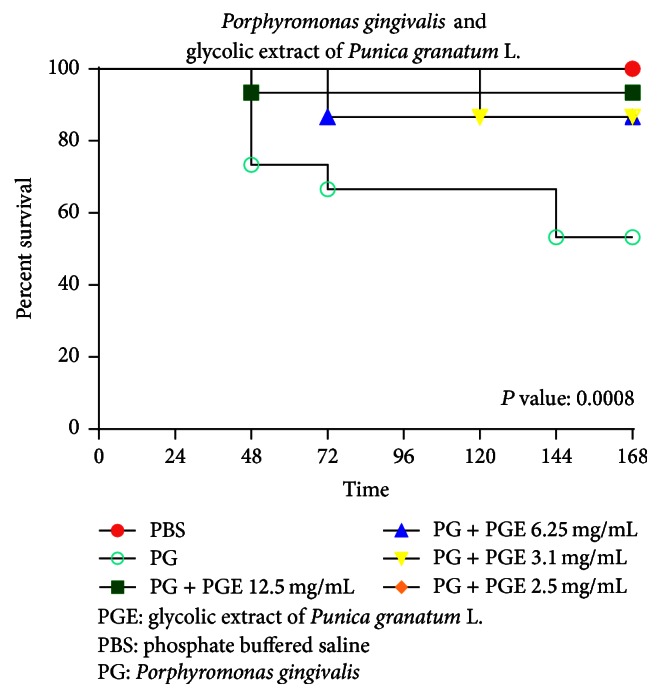
Survival curve of* G. mellonella* infected with* P. gingivalis* at different concentrations of pomegranate extract. The concentrations of PGE (12.5 mg/mL, 6.25 mg/mL, 3.1 mg/mL, and 2.5 mg/mL) diluted in PBS were inoculated in the last proleg of each larva. Fifteen larvae were used per group. The number of dead* G. mellonella* was daily recorded for 168 hours for analysis of the survival curve. The larvae survived at concentrations of 12.5 mg/mL, 6.25 mg/mL, 3.1 mg/mL, and 2.5 mg/mL.

The concentrations proving to be nonlethal to the larvae presented the following results when inoculated in* G. mellonella* infected by* P. gingivalis*: 12.5 mg/mL resulted in the death of 6% after 48 hours, 6.25 mg/mL resulted in the death of 13% after 72 hours, 3.1 mg/mL resulted in the death of 13% after 72 hours, and 2.5 mg/mL resulted in no death of* G. mellonella*. As shown in Figure 2, the control group (infected with* P. gingivalis* without any treatment) led to the death of 46% of the larvae after 48 hours.

## 4. Discussion

This study represents one of the first* in vivo* tests in* G. mellonella* assessing the activity of glycolic extract of* P. granatum *L. against* P. gingivalis*.* P. granatum *L. is indicated for many diseases, including treatment of caries, chronic and aggressive periodontitis, and gingivitis [14–16, 26] because it is rich in tannin [1, 26].

*P. gingivalis*, an opportunist Gram-negative anaerobic bacterium, plays a dominant role in both chronic and aggressive forms of periodontitis. Furthermore, subgingival colonization with high rates of this pathogen in the infected tissue has been demonstrated to increase the risk of disease progression [14]. The resistance of Gram-negative bacteria toward antibacterial substances is related to the hydrophilic surface of their outer membrane rich in lipopolysaccharide molecules, presenting a barrier to the penetration of antibacterial substances. Also, the enzymes in the periplasmic space are capable of breaking down the molecules introduced from outside [27, 28].

There are* in vitro* studies evaluating the efficacy of* P. granatum *L. extract against several microorganisms with cariogenic activity (Gram-positive bacteria, Gram-negative bacteria, and yeasts). One of these studies was performed by Abdollahzadeh et al. [1], in which different concentrations of methanolic extract of* P. granatum *L. promoted high inhibition of* Staphylococcus aureus* and* Staphylococcus epidermidis, *suggesting an antimicrobial action of this extract. This activity is also related to the presence of hydrolyzable/gallic tannins and polyphenols in the* P. granatum L* extract, such as punicalagin and gallic acid. These tannins can act on the bacteria's cell membrane, precipitating proteins and suppressing some enzymes. In turn, the polyphenols can affect the cellular wall by making some enzymes interact with proteins, thus impairing coaggregation of microorganisms [1].

In the present study, it was possible to find a high inhibition of the* P. gingivalis* activity following administration of glycolic extract of* P. granatum *L. As this extract consists of gallic tannins and alkaloids, one can suggest that these components may be accounting for the significant antimicrobial action against this Gram-negative bacterium. These results are corroborated by Dastjerdi et al. [29], who evaluated the effect of* P. granatum *L. water extract on 5 common oral bacteria (i.e.,* Streptococcus mutans*,* Streptococcus sanguinis*,* Streptococcus salivarius*,* Streptococcus sobrinus*, and* Enterococcus faecalis*) and then the magnitude of the inhibitory effect of* P. granatum *L. on bacterial biofilm formation on orthodontic wire. In this study, the following concentrations were used: 100 mg/mL, 50 mg/mL, 25 mg/mL, 12.5 mg/mL, and 6.25 mg/mL to 3.12 mg/mL. The minimum inhibitory concentration (MIC) and minimum bactericidal concentration (MBC) of this extract for* Streptococcus sanguinis* were 6.25 mg/mL and 25 mg/mL, respectively, which were the lowest MIC and MBC values. The extract was also able to reduce bacterial biofilm formation on orthodontic wire.

Maekawa et al. [18] assessed the antimicrobial action of dry, fresh, and glycolic extracts of* Zingiber officinale* against* Enterococcus faecalis* infection by using the* in vivo G. mellonella* model. The concentrations of the extract inoculated in* G. mellonella* larvae were 2.5 mg/mL, 5 mg/mL, and 2.5 mg/mL, respectively, for fresh, dry, and glycolic extracts. After survival analysis, all the experimental groups were shown to have a significant increase in the larval survival [18].

Based on these studies, the extract concentrations used in the present work were similar, that is, ranging from 200 mg/mL to 2.5 mg/mL. The concentrations of* P. granatum *L. extract at 12.5 mg/mL, 6.25 mg/mL, 3.1 mg/mL, and 2.5 mg/mL demonstrated antimicrobial action against* P. gingivalis* infection in the* G. mellonella* model. These results are corroborated by de Oliveira et al. [10], who observed that glycolic extract of* P. granatum *L. had an inhibitory activity on* Staphylococcus aureus*,* Staphylococcus epidermidis*,* Streptococcus mutans*,* Candida albicans*,* Candida tropicalis*, and* Candida glabrata*. The elimination of all strains of* Staphylococcus aureus* was determined by using a 12.5 mg/mL concentration, whereas concentrations of 6.25 mg/mL and 25 mg/mL were effective for eliminating all strains of both* Staphylococcus epidermidis* and* Streptococcus mutans* as well as of* Candida* spp., respectively [15].

Maekawa et al. [18], who tested the antimicrobial activity by using a glycolic extract of* Zingiber officinale*, and Argenta et al. [30], who used a glycolic extract of 3% pomegranate in dentifrices, found the same pattern of inhibition seen in the chlorhexidine on* Streptococcus mutans. *In addition to this study, the glycolic extracts prepared at 10% and 3% concentrations produced similar inhabitation halos of 18 mm.

In this way, the above-mentioned results demonstrated the antimicrobial action of* Punica granatum *L. from different means of extraction and using several parts of the plant or its fruit.

Based on several studies demonstrating the* in vitro* antibacterial activity of* P. granatum *L. and because of the scarcity of* in vivo* studies on* P. gingivalis*, our* in vivo* study demonstrated the antibacterial activity of the* P. granatum* L. extract on* P. gingivalis *in* G. mellonella*. In addition,* G. mellonella* can be a useful model to study* P. gingivalis* and potential antimicrobial agents for oral infections.

## 5. Conclusion

In conclusion, the glycolic extract of* P. granatum *L. had antimicrobial action against* P. gingivalis in vivo* model using* G. mellonella*.

## Figures and Tables

**Table 1:** Experimental groups.

Groups	*N*	Left proleg	Right proleg
*P. gingivalis *(positive control)	15	10 *μ*L *P. gingivalis*	—
*P. gingivalis* + PGE^*∗*^	15	10 *μ*L *P. gingivalis*	10 *μ*L PGE
PGE^*∗*^	15	10 *μ*L PGE	—
PBS (negative control)	15	10 *μ*L PBS	—

*Notes*: PGE: pomegranate glycolic extract; PBS: phosphate buffered saline.

^*∗*^Extract concentrations (12.5 mg/mL, 6.25 mg/mL, 3.1 mg/mL, and 2.5 mg/mL) not toxic to *G. mellonella*.
